# Agricultural nanodiagnostics for plant diseases: recent advances and challenges

**DOI:** 10.1039/c9na00724e

**Published:** 2020-07-06

**Authors:** Zheng Li, Tao Yu, Rajesh Paul, Jingyuan Fan, Yuming Yang, Qingshan Wei

**Affiliations:** Institute for Advanced Study, Shenzhen University Shenzhen 518060 P. R. China; Department of Chemical and Biomolecular Engineering, North Carolina State University 911 Partners Way, Campus Box 7905 Raleigh NC 27695 USA qwei3@ncsu.edu; Department of Polymer Science and Engineering, Zhejiang University Hangzhou 310027 P. R. China; Department of Agrotechnology and Food Sciences, Wageningen University 6708 PB Wageningen The Netherlands; Emerging Plant Disease and Global Food Security Cluster, North Carolina State University USA

## Abstract

Crop diseases caused by pathogenic microorganisms pose severe threats to the global food supply. Effective diagnostic tools for timely determination of plant diseases become essential to the assurance of agricultural sustainability and global food security. Nucleic acid- and antibody-based molecular assays are gold-standard methodologies for the diagnosis of plant diseases, but the analyzing procedures are complex and laborious. The prominent physical or chemical properties of nanomaterials have enabled their use as innovative and high-performance diagnostic tools for numerous plant pathogens and other important disease biomarkers. Engineered nanomaterials have been incorporated into traditional laboratory molecular assays or sequencing technologies that offer notable enhancement in sensitivity and selectivity. Meanwhile, nanostructure-supported noninvasive detection tools combined with portable imaging devices (*e.g.*, smartphones) have paved the way for fast and on-site diagnosis of plant diseases and long-term monitoring of plant health conditions, especially in resource-poor settings.

## Abstract 2

Engineered nanomaterials integrated with molecular assays or miniature sensing devices formed a promising nanodiagnostic tool box for plant diseases.

## Graphical abstract

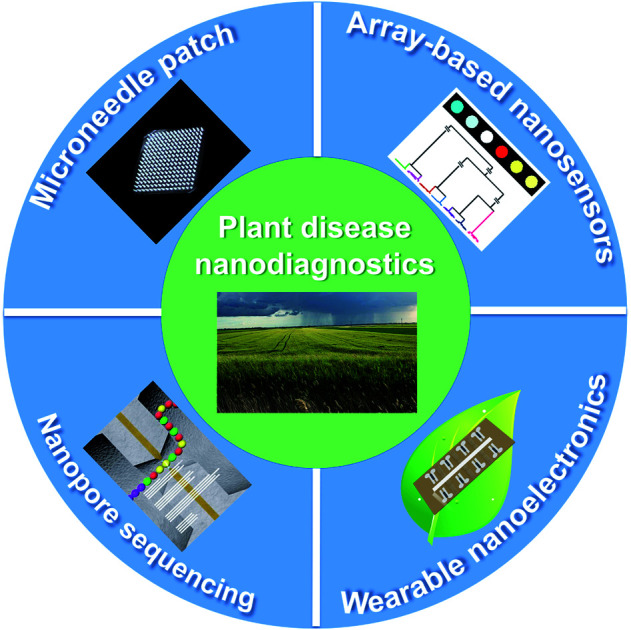

## 1. Introduction

Nowadays, over one billion people in the world are suffering from varying degrees of starvation due to the lack of basic staples and insufficient nutritional intake.^1^ This situation is largely attributed to the continuous increase of global populations, and reduction of agricultural productivity due to social, economic, and environmental reasons. Crop disease caused by pathogen infection and pest attack is one of the main constraints of agricultural production and has become one of the critical global issues.^2^ For example, the annual losses of main economic crops including potato, maize, peanut, and soybean due to pathogen infections are estimated to be 10–25% of total production.^3,4^ In order to reduce the spread of plant diseases and facilitate the management of most severe infections, the development of advanced diagnostic tools for early and precise determination of common plant diseases becomes essential.

Nanotechnology is one of the most intriguing and thriving areas of science, which has profound influence on a large number of subjects including science, engineering, medicine, and agriculture.^4,5^ Nanomaterials are ideal candidates for the analysis of plant pathogens as their dimensions typically fall in the range of 1 to 100 nm that can provide enhanced surface-to-volume ratio and unique chemical, optical, and electrical properties, which are not observed in the bulk counterparts. Nanoscale materials can also interact with biomolecular targets in a more efficient way due to their small sizes and fast diffusion rates. Nanomaterials can be prepared in many different morphologies ranging from spherical particles, cubes, rods, wires, plates, prisms, core–shell structures, to more complicated 3D architectures, which could undergo shape transformation or agglomeration that alters their chemical or physical properties in response to various external stimuli.^6^ This essentially becomes one of the most common sensing mechanisms of nanomaterials. Breakthroughs in nanotechnology achieved in the recent decade allow for the preparation of nanomaterials for a broad range of biosensing or bioimaging applications, especially those for healthcare monitoring.^7–9^ A wide range of micro- or nanostructured platforms or sensors have been developed and integrated into the standard molecular diagnostic toolbox that provides extraordinary sensitivity and spatiotemporal resolution.^10^ The field of agricultural biosensors is also emerging in the past few years.^11–14^ Due to their cost-effectiveness and field portability, nanosensors have already demonstrated tremendous promise for accurate detection of pathogens that cause severe infections of various economically important crops and plants.^15–19^

In this minireview, we focus on the emerging use of engineered nanomaterials as potential diagnostic tools in agricultural settings. The design and operation of a variety of nanomaterial-based sensors and detection platforms will be discussed. We will particularly examine studies from the last few years, where the most state-of-the-art nanosensors or nanostructures were reported as easy-to-use devices for plant disease diagnosis. Based on whether nanoparticles (referring to 0D, 1D, or 2D nanostructures with at least one dimension less than 100 nm) are actively used in the sensor devices, we generally divided emerging nanodiagnostic tools for agricultural applications into two main categories: (1) nanostructured devices and tools (*e.g.*, microneedle patch, nanopore, and wearable devices), and (2) nanoparticle-based sensors (*e.g.*, gold nanoparticle, quantum dot, and array-based nanosensors). Finally, insights will be given to the current challenges and future directions in the development of nanodiagnostic tools for plant health monitoring.

## 2. Methods for diagnosis of plant diseases

**Fig. 1:**
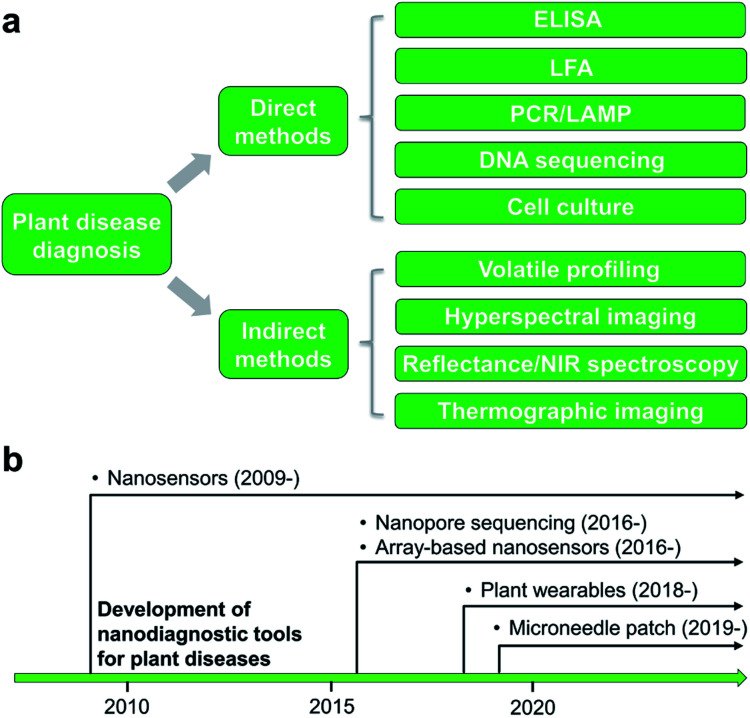
Methods for plant biotic stress monitoring. (a) Categories of different direct or indirect techniques for plant disease detection. (b) Chronological advances in nanoscale tools for plant disease diagnosis.

Detection and identification of plant diseases could be achieved by both direct and indirect methods.^20,21^ Direct approaches typically involve analysis of plant pathogens (*e.g.*, bacteria, oomycetes, fungi, and viruses) or biomolecular markers (*e.g.*, nucleic acids, proteins, and carbohydrates) isolated from infected plant tissues. Indirect diagnosis recognizes plant diseases through changes in physiological or histological indices such as leaf surface temperature or humidity, spectroscopic features of plant tissues, morphology, growth rate, and emissions of volatile organic compounds (VOCs). A wide variety of spectroscopic,^22,23^ electrochemical,^24^ or molecular technologies^25^ could serve as direct or indirect detection methods (Fig. 1a). This minireview will summarize several mainstream categories of nanoscale techniques for agricultural diagnosis, in both direct or indirect fashions, including microneedle patches, nanopore sequencing platform, plant wearables, and nanoparticle or array-based sensors. The development of nanodiagnostic tools for AgBio science is in its infancy (Fig. 1b). While many nanosensing tools have been previously prepared and demonstrated for human health monitoring, the applications of nanosensors to agriculture started relatively recently in around 2009. Other new sensing technologies such as nanopore sequencing and array-based nanosensors only moved into the AgBio area in around 2016, and the development of plant wearable and microneedle tools is even latter (Fig. 1b). In recent years, the adoption and application of new technologies to the field of agriculture and plant science has been even more accelerated. For example, the newly developed CRISPR technology has already found a wide range of applications in agriculture and food industry.^26^

### 2.1 Nanostructured devices and tools

#### 2.1.1 Microneedle patches

Conventional diagnostics in agriculture are based on nucleic acid amplification and protein-based molecular assays, such as enzyme-linked immunosorbent assay (ELISA),^27^ polymerase chain reaction (PCR),^28^ loop-mediated isothermal amplification (LAMP),^29–31^ and recombinase polymerase amplification (RPA).^32^ These measurements have been extensively carried out for routine laboratory analysis of biomolecules to identify specific pathogens and strains. Despite the high sensitivity and specificity toward particular pathogens, traditional molecular diagnostic tests suffer from several major drawbacks such as complicated sample preparation, long assay time, and expensive instrument, which limit their application and use in the field.^33^ The integration of nanotechnology with standard biomolecular analyses could possibly provide a solution to improving sensitivity, portability, and versatility of traditional diagnostics that are often performed in centralized laboratories.^10^

**Fig. 2:**
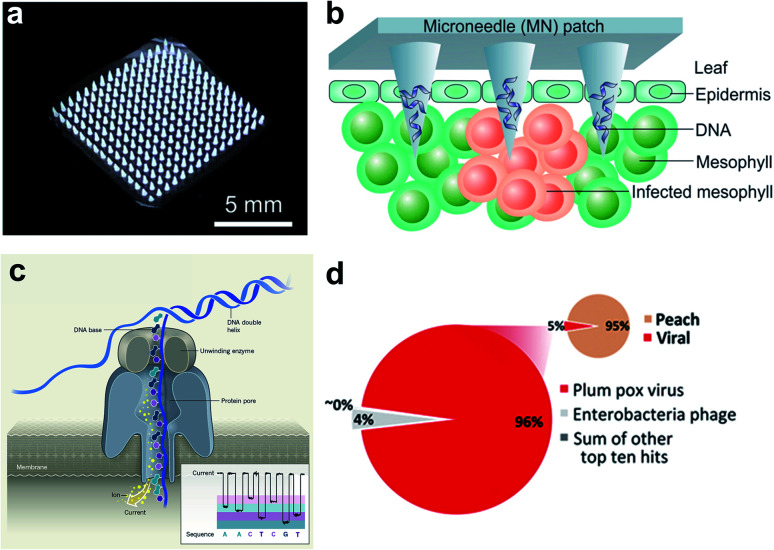
Microneedle-based plant DNA extraction and nanopore sequencing platform. (a) Photograph of a polymeric microneedle patch. (b) Illustration of plant pathogen DNA (*Phytophthora infestans*) extraction by applying MN patches on plant leaves. The microneedles can penetrate the leaf tissue and absorbs DNA on the surface of tips. (c) Principle of the handheld nanopore sequencing device (MinION) developed by Oxford Nanopore Technologies. (d) Alignment of reads produced by MinION from *Prunus persica* (peach) datasets to the total viral genomes using nanopore sequencing of whole transcriptome amplification. The target viruses are *plum pox virus* (PPV) and *Candidatus Liberibacter asiaticus*, two major viral pathogens of stone fruits. The inset shows the number of total viral reads *versus* the number of reads mapping to the *P. persica* genome. Figure panels reproduced from ref. 36 with permission from American Chemical Society, copyright 2019; ref. 47 with permission from American Phytopathological Society, copyright 2018.

Micro-analytical devices such as skin-penetrating microneedle (MN) patches are attractive for healthcare applications due to their high efficiency in noninvasive delivery of drug molecules, extraction of diagnostic analytes, and *in situ* monitoring of key clinical parameters. Although the size of the microneedles is typically in the micron scale (*e.g.*, 5–10 μm tip diameter), the needle matrix can be loaded with functional nanoparticles or engineered with nanoporous structures to enhance sensing and sample interaction (*e.g.*, fluid extraction). Microneedle patches have been extensively employed in nanomedicine for transdermal drug delivery^34^ and subdermal biosensing^35^ in human patients. However, few studies are available for point-of-care (POC) diagnosis of plant diseases. Recently, we demonstrated a polymer-based microneedle patch in combination with real-time PCR (*i.e.*, quantitative PCR, or qPCR) technique for rapid and sensitive detection of *P. infestans*, an oomycete that causes the serious potato and tomato disease known as late blight (Fig. 2a and b).^36,37^ One of the major challenges for on-site plant disease detection through molecular assays is to isolate specific biomarkers from robust and rigid plant tissues. To overcome this obstacle, a prototype microneedle patch made of polyvinyl alcohol (PVA) was developed that is sufficiently strong to break the cell walls of plant leaf tissues to release plant or pathogenic DNA.^36,37^ The developed DNA extraction method is simple enough to reduce sample preparation time from several hours to less than 1 min. The isolated DNA is ready for amplification without the need for any additional sample purification steps. Despite the lack of specificity in isolation of nucleic acids and other intracellular analytes in the current microneedle design, integration of effective microneedle-patch techniques with miniaturized DNA amplification assays^38,39^ opens up a path for facile, rapid, and field-deployable amplification and diagnosis of plant pathogens.

#### 2.1.2 Nanopore sequencing platform

The development of single-molecule sequencing technology has brought about impressive advancements in the detection accuracy and sensitivity of probing DNA structure variations for both clinical and agricultural studies.^40,41^ As a third-generation sequencing (TGS) technique, the nanopore sequencing platform uses a motor protein to transport a single-stranded DNA or RNA through either a protein or a solid-state nanopore to generate nucleotide-dependent electronic current signals (Fig. 2c),^42^ which enables facile analysis of pathogenic polynucleotides. Compared to earlier generations of sequencing techniques, nanopore sequencing platform takes advantages of real-time and long reads of sequence data, fast running times, scalable genome mapping, and small sample loadings.^43–46^

A large number of latest studies using nanopore sequencing platforms have involved in the plant pathogen diagnosis. For example, Chalupowicz *et al.* have successfully set up a standard protocol using a handheld sequencing system developed by Oxford Nanopore Technologies (named “MinION”) for diagnosis of different plant bacteria, viruses, fungi, and phytoplasma, including *P. digitatum* in lemon and *S. lycopersicum* in tomato. The total assay time is within 2 h, and the results are comparable to conventional diagnostic procedures (*e.g.*, PCR and ELISA).^46^ Badial *et al.* demonstrated the capability of nanopore sequencing coupled with whole transcriptome amplification for relatively rapid detection (within 24 h) of two viral species, *Candidatus Liberibacter asiaticus* and *plum pox virus*, in the peach (Fig. 2d).^47^ Filloux *et al.* reported high genome mapping results achieved by MinION by predicting the presence of several plant virus species, including *Dioscorea bacilliform virus*, *Yam mild mosaic virus* and *Yam chlorotic necrosis virus*, in a water yam plant.^48^ Despite some challenges remaining in the present equipment such as high per-read error rate and poor discriminatory ability among similar sequences,^48^ continuous breakthroughs in nanopore technology will lead to the creation of more powerful sequencing platforms.

#### 2.1.3 Plant wearables

**Fig. 3:**
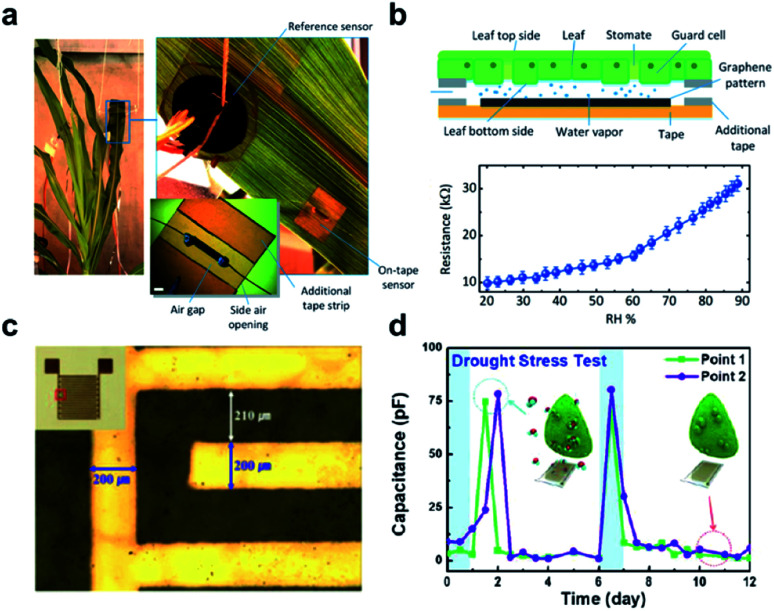
Wearable sensors for the monitoring of plant health. (a) A photograph of the graphene-based relative humidity (RH) sensor for the detection of water movement from the roots to the lower and upper leaves within the plant. (b) Schematic illustration of the construction and detection mechanism of the RH sensor (upper figure). The lower figure shows the resistance measurement of this sensor as a function of RH. (c) Optical image and real image (inset) of a flexible polyimide-based plant drought sensor. (d) The drought stress responses over time on the Nicotiana tabacum leaf. The blue arrow points to normal conditions, while a red arrow points to drought. Figure panels reproduced from ref. 49 with permission from Wiley, copyright 2017; ref. 51 with permission from MDPI, copyright 2018.

With recent advances in the technology of micro-electro-mechanical systems (MEMS), more and more implantable and wearable electronics using conductive nanomaterials have emerged as sensor components for long-term and on-demand monitoring of plant VOCs or other biomarkers. Plant wearable becomes a new frontier of crop diagnostics, which relies on ultrathin and ultra-lightweight nanosensor design to attach flexible sensor devices directly on plant tissues such as leaves for continuous monitoring. For example, Oren *et al.* developed a roll-to-roll fabrication method of a graphene-based wearable sensor that can monitor water evaporation from plant leaves (Fig. 3a and b).^49^ The sensing mechanism is based on changes in the electrical resistance of graphene in different humidity environments. Im *et al.* reported a wearable plant drought stress sensor based on a different mechanism, where the variation of the capacitance of printed 100 nm-think gold electrodes on a flexible polyimide (PI) was recorded in real-time to monitor local humidity.^50^ Nassar *et al.* developed a lightweight and multiplexed plant wearable that integrates temperature (resistance), humidity (capacitance), and strain sensors (resistance) to monitor plant's local microclimate and plant growth (Fig. 3c and d).^51^ The butterfly-shaped multisensory platform was fabricated by transfer printing of 180 nm thick gold electrodes onto flexible polyimide (PI)/polydimethylsiloxane (PDMS) substrate. The same group also demonstrated a flexible CMOS-enabled sensor platform that is equipped with a bare die battery, microcontroller, and a bare die chip for simultaneous light, temperature, and humidity measurement.^52^ Lei *et al.* have demonstrated disintegrable and biocompatible pseudo-CMOS flexible circuits that can be attached to the rough surface of plants such as an avocado.^53^ Kim *et al.* developed a technique to prepare vapor-printed polymer electrodes, which can be directly printed on living plant tissues for long-term monitoring of drought and photodamage.^54^

### 2.2 Nanoparticle-based sensors

Imaging techniques, such as thermographic and hyperspectral imaging, have been used in the field for indirect detection of plant disease. However, many of them have notable limitations, such as susceptibility to parameter changes of the environment and lack of specificity for disease subtypes or strains.^55,56^ Rapid progresses in chemo- or biosensing technology have led to a broad range of successful applications, such as quality assessment of medicinal and industrial products.^57^ Recently, several nanoparticle-based chemo- or biosensors have been proposed and commercialized for agricultural diagnosis.^11,13,58,59^ Depending on transduction mechanisms of the designed sensory interactions, the analytes could be recognized by characteristic optical or electrical outputs of the nanosensors. The sensor's detection specificity could be enhanced by selective chemical interactions or by the use of biospecific recognition elements such as DNA oligos, antibodies, aptamers, and enzymes. The detection sensitivity could be improved by the use of surface-enhanced optical properties (*e.g.*, surface plasmon resonance (SPR)) or electron-conductive nanoscale substrates such as carbon-based nanomaterials (*e.g.*, graphene or carbon nanotube) as transducers.^60^ In this section, we will highlight three main classes of nanosensors or nanobiosensors, including metal or metalloid nanoparticles, quantum dots, and array-based nanosensors. A list of representative nanosensors or biosensors used for plant disease diagnosis, their detection mechanisms, and associated performance is summarized in Table 1.

**Table 1:** Summary of nanoparticle-based sensors for plant pathogen or disease marker detection

Target	Sensor component	Sensor Fabrication	Detection mechanism	Sensitivity	Specificity	Toxicity	Ref.
*P. syringae*	Au NP^a^-ssDNA	Nanoparticle functionalization and DNA hybridization	Electrochemistry	214 pM	Unresponsive to *B. cinerea* and *F. oxysporum*	Low	62
*P. ramorum* and *P*. lateralis	Ag NP-ssDNA	Nanoparticle functionalization and DNA hybridization	SERS^b^	N/A	Unresponsive to *P. lateralis*	Low	64
Aflatoxins	Ag NR^c^	Surface functionalization	SERS	5 × 10^−5^ M for Aflatoxin B1	Discriminable among Aflatoxin B1, B1, G1 and G2	Low	66
*B. thuringiensis* and *B. subtilis*	Pt NP-IgG^d^	Surface immunological functionalization	MALDI-TOF MS^e^	∼10^2^ CFU	Discriminable between two bacteria	Low	67
*X. campestris*	Si NP-Rubpy-IgG	Surface immunological functionalization	Fluorescence quenching	3 × 10^2^ CFU	N/A	Moderate	68
*R. solanacearum*	Au NP-ssDNA	Surface functionalization	Colorimetry	7.5 ng	No specific band revealed by PCR	Low	69
*F. oxysporum*	CdSe/ZnS-MPA^f^ QD^g^	Surface functionalization	Fluorescence	25 μg mL^−1^	N/A	High	73
*Arabidopsis thaliana*	CdSe-PEI^h^ QD	Surface functionalization	Fluorescence	10 μg mL^−1^	N/A	High	75
*V. mali*	CD^i^	Surface functionalization	Fluorescence	pH range of 2.13–9.34	No interference from ions	Low	79
*F. avenaceum*	CD	Surface functionalization	Fluorescence	N/A	Discriminable between *P. aeruginosa* and *F. avenaceum*	Low	80
3′-Diphosphate-5′-diphosphate	Tb(iii)-CD	Metal ligation	Fluorescent ratiometry	50 nM	Unresponsive to analogues including ATP, CTP, and UTP	Moderate	81
*Citrus tristeza*	CdTe QD-CD	Surface immunological functionalization	FRET^j^	520 ng mL^−1^	N/A	High	84
*Citrus tristeza*	CdTe QD-Rd^k^	Surface immunological functionalization	FRET	220 ng mL^−1^	N/A	High	85
*p*-Ethylguaiacol	TiO_2_ and SnO_2_ NP	Surface functionalization	Electrochemistry	35–62 nM	Slightly responsive to *p*-ethylphenol, unresponsive to 3-octanone and 1-octen-3-ol	Low	96
Ethylene	Cu(i) tris(pyrazolyl) borate-SWCNT^l^	Ball-milling	Chemiresistor	0.5 ppm	Unresponsive to common solvents except acetonitrile, THF, and acetaldehyde	Low	99
Ethylene	Pd(ii)-pH dye-SiO_2_ microspheres	Ultrasonic spray pyrolysis	Colorimetry	0.17 ppm	Unresponsive to alcohols or esters	Moderate	100
Terpene vapor	Au NP@sol–gel	One-pot hydrolysis	LSPR	N/A	Discriminable among *cis*-jasmone, α-pinene, limonene, and γ-terpiene	Low	101
Various VOCs^m^	Au NR-cysteine and nanoporous dyes	Surface functionalization	LSPR	0.4–1.7 ppm for hexenal; 1.8–5.2 ppm for phenol	Discriminable among over ten VOCs	Low	102

a Nanoparticle.

b Surface-enhanced Raman spectroscopy.

c Tris(bipyridine)ruthenium(ii) chloride.

d Immunoglobulin G.

e Matrix-assisted laser desorption/ionization-time of flight mass spectroscopy.

f 3-Mercaptopropionic acid.

g Quantum dot.

h Polyethylenimine.

i Carbon dot.

j Förster resonance energy transfer.

k Rhodamine 123.

l Single-walled carbon nanotube.

m Volatile organic compounds.

#### 2.2.1 Metal or metalloid nanoparticles

**Fig. 4:**
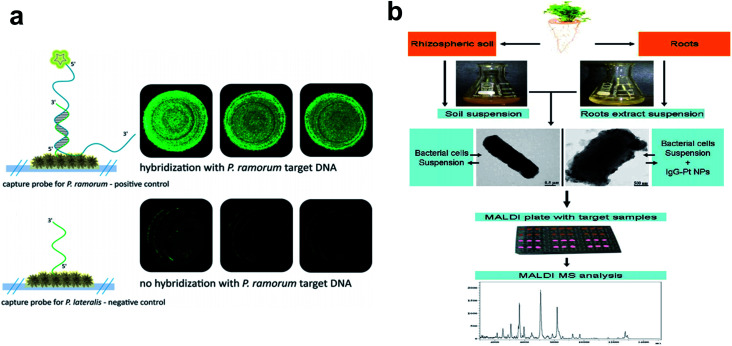
Metallic nanoparticles as nanobiosensors in plant pathogen detection. (a) Fluorescence microscopy to verify the functionality of the hybridization assay on an Ag nanoparticle-based SERS substrate. Top: hybridization of *P. ramorum* target DNA with matching *P. ramorum* capture probes; bottom: absence of signals for *P. ramorum* target DNA with non-matching *P. lateralis* capture probes. (b) Workflow chart showing procedures of Pt nanoparticle-assisted MALDI MS analysis of plant-associated bacteria from soil and root samples. Figure panels reproduced from ref. 68 with permission from Royal Society of Chemistry, copyright 2015; ref. 71 with permission from Elsevier, copyright 2012.

Inorganic nanomaterials such as Ag, Au, Si, and various metal oxide nanoparticles form a common class of nanosensors that use recognition ligands (*e.g.*, antibodies or DNA oligos) and signal transducers to detect and quantify molecular targets of interest.^61,62^ Those nanoparticles can serve as unique sensing platforms for probing interactions between the nanoparticles and bioanalytes of specific relevance to plant diseases *in vitro* or in living plant systems.^63^ Characteristic sensor responses are often recorded through changes in the surfaced-enhanced optical properties, originating from the localized surface plasmon resonance (LSPR) of nanoparticles. A myriad of self-assembled nanoparticles with oligonucleotides^64–66^ or proteins^67^ have been explored to construct sensitive and target-specific nanobiosensors. For example, Ag nanorods have been frequently employed as sensor substrates for the identification of various plant pathogens or produced toxins using surface-enhanced Raman spectroscopy (SERS),^68–70^ as shown in Fig. 4a. Pt nanosensors functionalized with IgG antibodies were introduced to matrix-assisted laser desorption/ionization mass spectrometry (MALDI MS) that greatly improved the sensitivity to detect plant-associated bacteria in soil and carrot (Fig. 4b).^71^ Fluorescent silica nanoparticles doped with a Ru(ii) complex and conjugated with a secondary antibody were reported to successfully detect plant pathogens such as *X. campestris* that caused bacterial spot disease in nightshade plant.^72^ The method was highly sensitive, but the environmental concerns regarding the use of a potentially hazardous heavy metal complex were not addressed. Au nanoparticles functionalized with a specific single-stranded DNA were used to detect as low as 15 ng of genomic DNA of *R. solanacearum*, a soil-borne species that could cause potato bacterial wilt.^73^ The assay showed sufficiently high specificity toward the target DNA, yet more quantitative calibration results at the lower detectable range were unavailable.

Nanosensors or nanobiosensors also play vital roles in detecting and controlling the use of pesticides, fertilizers, as well as many other growth parameters associated with crops, which provide timely information for precise decision making and agricultural management.^74^ Nevertheless, more field validation tests are needed for sensor applications in precision farming.

#### 2.2.2 Quantum dots

**Fig. 5:**
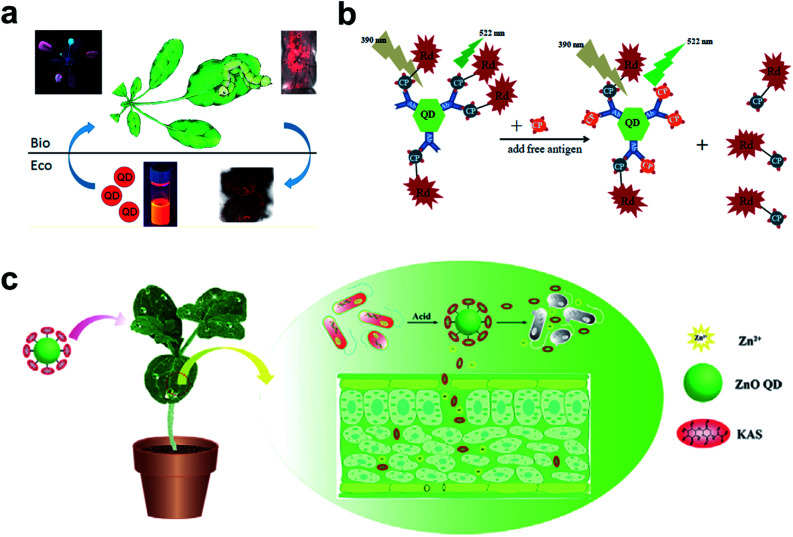
QDs used in the plant system. (a) Fluorescence imaging of the uptake and transport of QDs in *Arabidopsis thaliana*. (b) Schematic illustration of specific CTV biosensor based on FRET. (c) ZnO QDs were used to conjugate KAS for the controlled release of pesticides for the plant system. Figure panels reproduced from ref. 79 with permission from American Chemical Society, copyright 2015; ref. 89 with permission from International Frequency Sensor Association Publishing, copyright 2017; ref. 90 with permission from Elsevier, copyright 2019.

Quantum dots (QDs) are semiconductor nanocrystals that have shown tremendous potentials as optical nanosensors.^75^ Owing to their unique and advantageous photophysical properties, QDs have found successful applications as biosensors for plant imaging and disease identification.^76^ The ultra-small size of the QDs (1–10 nm) makes them ideal fluorescent imaging contrast agents for rapid in-planta uptake and transport, where the fluorescent signals can be readily captured to track their distributions in the living system (Fig. 5a). Current studies regarding plant pathogen detection were typically performed *in vitro*. Prats *et al.* reported a CdSe–ZnS core–shell QD with 3-mercaptopropionic acid coating, which could be quickly taken up by fungal hyphae.^77^ Despite an increasing number of endeavors on QD uptake by different model plant species, the interactive/delivery mechanisms and underlying side effects of QDs yet remains to be investigated.^78,79^

As a competitive alternative to conventional semiconductor QDs, the newly emerging carbon dots (CDs) have attracted wide attention largely due to the ease of preparation from fruits^80^ and vegetables.^81^ The low cytotoxicity and high biocompatibility make them valuable in bacterial and fungal imaging.^82–84^ Huang *et al.* developed a paper-based, fluorescent Tb(iii)-CD probe to detect 3′-diphosphate-5′-diphosphate, the stringent of plants in response to extreme environmental conditions. The limit of detection (LOD) was down to 50 nM based on the synergistic effect between Tb(iii) ions and CDs.^85^ In addition to inherent fluorescence enhancement or quenching, fluorescence resonance energy transfer (FRET)^86^ is another well-established mechanism for the construction of QD sensors. For FRET sensors, the proximity of the donors (*i.e.*, QDs) to acceptors (*e.g.*, gold NPs,^87^ carbon nanodots,^88^ and organic dyes^89^) leads to an energy transfer, which results in quenched fluorescence intensity. Safarnejad *et al.* reported a FRET-based complex sensor for probing *Citrus tristeza virus* (CTV).^89^ To achieve this goal, CdTe QDs conjugated with CTV coat protein (CP) and CP-labelled rhodamine dye were employed as donor–acceptor complexes. The presence of target viruses resulted in the replacement of CP-rhodamine with free CP, rendering a recovered fluorescence of QDs (Fig. 5b).

Furthermore, surface-functionalized QDs can be employed as on-demand agrochemicals for plant disease management. For example, kasugamycin (KAS), a kind of natural antibiotic, was artificially conjugated to the surface of ZnO QDs.^90^ The prepared KAS-ZnO QDs showed outstanding pH-responsive property and improved photostability. In the greenhouse experiment, KAS and Zn(ii) species could be released in a controlled manner, which significantly alleviated the severity of bacterial fruit blotch (Fig. 5c).

Due to the extensive use of transition metal elements as optical nanoprobes, issues associated with their toxicity become increasingly inevitable. For field applications where diagnostic efficiency is of high priority, health risks of quantum dots could be overlooked since many of them are made of highly toxic heavy metals (Pb, Zn, and Hg).^91,92^ Using environmentally benign elements such as carbon or silica could potentially relieve this negative effect. Another concern for optical imaging of plant biomarkers using nanoparticles lies in the possible interferences from autofluorescence of host plant tissues due to ubiquity of biological pigments (*e.g.*, chlorophyll or carotenoid) in plants that may have similar emission bands.^93^ Therefore, rational selection of excitation/emission wavelengths of engineered nanosensors is critical to the acquisition of explicit spectral information about targets in a plant body.

#### 2.2.3 Array-based nanosensors

Among the state-of-the-art nanosensors, one format capable of multiplexing and discrimination of different analytes is the array-based sensor assemblies composed of several chromophores or synthetic nanomaterials as an analog to the mammalian olfactory system. A common form of the array-based sensor is also known as the “electronic nose” (e-nose), which incorporates electronic transducers instead of chemical sensors in the array.^94–96^ The array-based approach is particularly beneficial in distinguishing highly similar analyte mixtures due to its cross-reactivity and ability to measure molecular fingerprints. The multidimensional output data of sensor arrays can be analyzed by supervised or unsupervised chemometrics, which provides highly precise and predictive results.^97^ Alternatively, array-based chemical sensors^98^ can provide optical readout (*e.g.*, colorimetric or fluorescent), which is easier to analyze and interpret compared with e-nose signals. In addition, chemical sensor arrays are easy to fabricate, based on specific chemical interaction, which brings in much better chemical specificity, extremely cost-effective, and more resistant to environmental interference such as humidity and temperature than most e-nose devices. Chemical sensor arrays can be easily prepared by embedding various analyte-responsive dyes such as Lewis acid-base colorants, Brønsted acidic or basic colorants, solvatochromic or vapochromic dyes, and redox indicator colorants in hydrophobic nanoporous substrates such as modified silica sol–gels.^96^

**Fig. 6:**
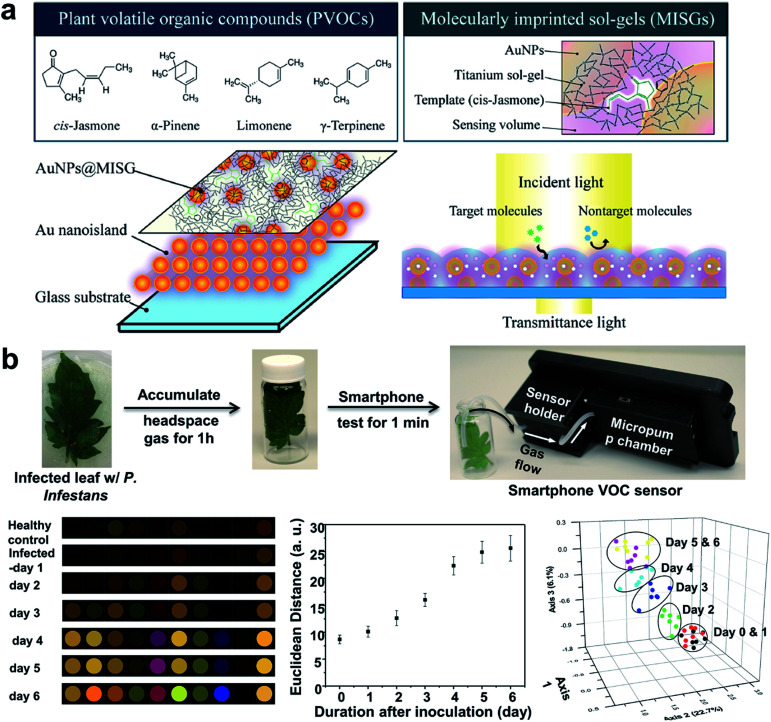
Different array-based nanosensor platforms for plant monitoring. (a) Schematic of AuNPs@MISG-coated Au nanoislands for selective detection of terpenes. (b) VOC sampling and detection of tomato late blight enabled by a 10-element nanostructured colorimetric sensor array using a smartphone-based detector. Early infection can be detected by the smartphone platform 2 days after inoculation. Figure panels reproduced from ref. 106 with permission from American Chemical Society, copyright 2018; ref. 107 with permission from Springer, copyright 2019.

An important indirect method for plant disease detection involves the profiling of the volatile chemical signature of diseased plants.^99^ This is based on the observations that diseased plants could result in the release or change of the composition of characteristic volatile organic compounds (VOCs) that are indicative of the type of biotic or abiotic stresses the host plants have experienced.^100^ E-noses made of metal oxides^101^ or conductive polymer coatings have drawn considerable attention as multiplexed gas sensors for tracing VOC biomarkers in plant stress events ranging from pathogen infection,^101^ pest invasion,^102^ to physical wounding.^103^ Particular emphasis is placed on the detection of ethylene, one of the most important phytohormones, using chemiresistive^104^ or colorimetric method.^105^ Of special interest is the design of a nanoplasmonic sensor array comprising AuNPs and a molecularly imprinted sol–gel (AuNPs@MISG) for selective detection of terpenes (Fig. 6a).^106^ Recently, we developed a smartphone-integrated VOC sensing platform that utilized plasmonic nanoparticles for early detection and differentiation of tomato late blight (within 2 days of infection) from the other fungal pathogens that lead to similar symptoms of tomato foliage (Fig. 6b).^107^ The gold nanoparticles were functionalized with chemical ligands that were reactive to specific leafy VOCs, and printed on paper strips to generate unique patterns of color changes for each VOC. The results were then scanned by a smartphone microscope for digital pattern recognition and differentiation. The described sensor platform will be not only useful for fingerprinting plant pathogens of interest, but also well-suited for distinguishing various abiotic stresses, such as mechanical damages, drought, and nutrition deficiency. Future research may be focused on improving sampling protocols that do not require pre-collection and concentration of VOCs prior to data analysis.

Portable vapor analyzers embedded with gas chromatography (GC) tandem mass spectroscopy (MS) or ion mobility spectroscopy (IMS) represent another class of mobile tools for on-site analysis of plant health status that can determine the identity and abundance of each emitted VOC species.^108–110^ Due to facile device fabrication and fast target sampling, hand-held MEMS devices integrated with miniaturized GC-MS/IMS sensors are among the most effective, non-optical methods for plant health monitoring.

## 3. Conclusions, challenges, and perspectives

Nanoscale materials are promising candidates for plant disease detection due to the remarkable biospecificity of engineered molecular recognitions at the nanoscale, which has witnessed unprecedented development in the recent decade in combination with modern analytical techniques, including fluorescence microscopy, spectroscopic measurement, wearable sensors, and smartphone-based microscopy. In this minireview, we have highlighted the latest and most notable nanodiagnostic systems for plant disease detection in the laboratory or field settings. Owing to the rapid advances in nanotechnology and modern nanofabrication techniques in the recent decade, great progress in a variety of useful sensors, biosensors, and nanostructured platforms has been continuously emerging for plant disease analysis. One of the immediate impacts of these newly developed nanodiagnostic tools is that precision plant disease detection now becomes more accessible to the field workers or farmers. For example, many conventional laboratory tests such as nucleic acid amplification, sequencing, and VOC analysis now can be potentially performed directly in the crop field in a much faster and cost-effective fashion, due to the recent innovation of rapid plant DNA extraction technology enabled by microneedles, miniature DNA sequencing chips, and smartphone-based VOC sensors. Taking advantages of design flexibility, chemical diversity, multiplexity, and cost-effectiveness of nanomaterials, it is expected that more and more powerful nanoscale sensors and probes integrated with multimodal detection mechanisms will be developed that allow for quick detection and determination of infections caused by plant pathogens, as well as many other biotic or abiotic stresses.

However, currently available plant diagnostic tools still face three major challenges: (1) environmental impact and toxicity of engineered nanomaterials; (2) promptness of data sharing and disease forecasting; and (3) long-term sensor stability in extreme scenarios such as cold or hot weather, intensive sun exposure, and heavy wear. For the first challenge, before any nanosensors can be commercialized and deployed to the field, their safety concerns must be addressed as some of the nanoparticles such as QDs could be toxic. In particle, for nanosensors that will be left on live plants or consumable agricultural and food products, more careful toxicity testing and regulation are needed, as harmful nanomaterial residuals could potentially enter the food chain and be uptaken by the end users.

For the second concern, since the foremost prerequisite of disease diagnosis is always the timely report and forecast of infection events on site, the new generation of nanosensors are expected to be more wirelessly connected that can provide near real-time measurement. For example, continuous monitoring of VOC emission of plants is expected to provide more time dynamic information than conventional single-point measurements and therefore enable more accurate monitoring of plant stresses. In this regard, the recent development of field-portable sensor tools such as smartphone devices or plant wearables brings promisingly new opportunities to the *in situ* analysis of pathogens in the field by sharing and transmitting data almost in real-time. In order to support continuous measurement, sensor miniaturization, wireless data transmission, and integration with computational data processing pipelines such as machine learning and artificial intelligence (AI) will be among the critical areas to be addressed further.

Lastly, more durable and robust sensors that can withstand varied environmental conditions (*e.g.*, temperature, humidity, air pollutions, *etc.*) in the crop field are anticipated before any sensors can be deployed to the real field. This requires more fundamental research on the novel sensor materials, such as environmental-resistant substrates and nanoparticles. For example, volatile sensor arrays made of more durable materials, such as resilient paper or polymer substrates and photo-resistive dyes, will be needed for the field crops or sentinel plants for long-term monitoring, where the sensor signals could be regularly scanned by the mobile phone readers or wirelessly read out by electrical wearable devices. The latter holds great promise for monitoring of early infections in a large scale by remote and continuous measurement of relevant biomarkers from symptomless field plants.

Despite the remaining challenges, the recent development of miniature and cost-effective nanodiagnostic tools has shown tremendous potentials in improving plant disease diagnosis, management, and crop health monitoring in the long run. The future of AgBio sensors is indeed very bright in the coming era of digital farm and precision agriculture.

## Conflicts of interest

The authors declare no conflicts of interest.

## Supplementary Material
